# Ara h 1 CD4+ T cell epitope-based peptides: candidates for a peanut allergy therapeutic

**DOI:** 10.1111/cea.12113

**Published:** 2013-05-28

**Authors:** S R Prickett, A L Voskamp, T Phan, A Dacumos-Hill, S I Mannering, J M Rolland, R E O'Hehir

**Affiliations:** 1 Department of Immunology, Monash UniversityMelbourne, Vic., Australia; 2 Department of Allergy, Immunology and Respiratory Medicine, The Alfred Hospital and Monash UniversityMelbourne, Vic., Australia; 3 Immunology and Diabetes, St Vincent's Institute of Medical ResearchMelbourne, Vic., Australia

**Keywords:** Ara h 1, CD4^+^ T cell, Immunotherapy, peanut allergy, peptide, T cell epitope

## Abstract

**Background:**

Peanut allergy is a life-threatening condition; there is currently no cure. While whole allergen extracts are used for specific immunotherapy for many allergies, they can cause severe reactions and even fatalities in peanut allergy.

**Objective:**

To identify short, HLA-degenerate CD4^+^ T cell epitope-based peptides of the major peanut allergen Ara h 1 that target allergen-specific T cells without causing IgE-mediated inflammatory cell activation, as candidates for safe peanut-specific immunotherapy.

**Methods:**

Ara h 1-specific CD4^+^ T cell lines (TCL) were generated from peripheral blood mononuclear cells (PBMC) of peanut-allergic subjects using CFSE-based methodology. T cell epitopes were identified using CFSE and thymidine-based proliferation assays. Epitope HLA-restriction was investigated using blocking antibodies, HLA-genotyping and epitope prediction algorithms. Functional peanut-specific IgE reactivity to peptides was assessed by basophil activation assay.

**Results:**

A total of 145 Ara h 1-specific TCL were generated from 18 HLA-diverse peanut-allergic subjects. The TCL recognized 20-mer peptides throughout Ara h 1. Nine 20-mers containing the most frequently recognized epitopes were selected and their recognition confirmed in 18 additional peanut-allergic subjects. Ten core epitopes were mapped within these 20-mers. These were HLA-DQ and/or HLA–DR restricted, with each presented on at least two different HLA-molecules. Seven short (≤ 20 aa) non-basophil-reactive peptides encompassing all core epitopes were designed and validated in peanut-allergic donor PBMC T cell assays.

**Conclusions and Clinical Relevance:**

Short CD4^+^ T cell epitope-based Ara h 1 peptides were identified as novel candidates for a safe, T cell targeted peanut-specific immunotherapy for HLA-diverse populations.

## Introduction

Peanut allergy is the leading cause of food-induced anaphylactic fatalities world-wide 1, 2. It is a major health care problem affecting 1–2% of the population 2–4, with clinical symptoms ranging from mild oropharyngeal irritation to life-threatening anaphylaxis. Unlike egg and milk food allergy that is present in infants and typically resolves by school age, peanut allergy is lifelong in 80% of the cases. This significantly impairs the quality of life of afflicted individuals and their families 5–7, with further impact on the wider community through efforts to manage this severe condition 2, 8. There is currently no cure for peanut allergy. Avoidance is the only means of control, with epinephrine as emergency treatment for anaphylaxis. Even with diligent precautions, most peanut-allergic subjects have accidental exposures which can have severe or even fatal consequences 1, 4, 5, 9.

Whole allergen extracts are currently used for specific immunotherapy for respiratory and insect venom allergies, but are unavailable in clinical practice for treatment of food allergy due to risks of severe side-effects or even death in the case of peanut allergy. The limited studies on specific immunotherapy for peanut allergy provide encouragement that desensitization is feasible, but the observed adverse reactions highlight major safety concerns 10–18. These risks are especially pertinent for peanut allergy since peanut allergens may induce anaphylaxis at minute doses with little correlation between previous severity of reactions and a person's first anaphylactic episode 11, 19. Consequently, there is an urgent need to develop a safe, disease-modifying therapeutic for peanut-allergic individuals.

Anaphylaxis results from the release of inflammatory cell mediators triggered by binding and cross-linking of cell-bound allergen-specific IgE by the relevant allergen. During specific immunotherapy, stimulation of appropriate T cell responses is considered essential for successful desensitization and the subsequent reduction and/or inhibition of allergen-specific IgE 20–22. Although conventional immunotherapy administers whole allergen extracts, studies on cat allergy 23–27 and bee venom allergy 28, 29 clearly demonstrate that short T cell epitope-based peptides of major allergens are sufficient for effective desensitization without causing adverse IgE-mediated reactions. Importantly, targeting T cells specific for immunodominant epitopes of major allergens can alter responses to whole allergen extracts (linked suppression). Many studies reporting successful peptide immunotherapy in murine models of allergy demonstrated that administration of immunodominant T cell epitope peptides of major allergens induced tolerance not only to those peptides but also to purified allergen and whole allergen extracts 30–35. More recently, clinical administration of Fel d 1 T cell epitope peptides in humans altered T cell responses to those peptides, other non-related Fel d 1 peptides and whole cat allergen extract 25.

We aimed to design peptides based on the most reliably recognized CD4^+^ T cell epitopes of major peanut allergens for a T cell-targeted immunotherapy for peanut allergy as a safe (non-IgE reactive) and effective alternative to whole allergens. Of 11 peanut allergens identified (Ara h 1-11) [Bibr b36], Ara h 1 and Ara h 2 are the two designated major allergens whose recognition is most consistently reported in > 50% of cohorts tested 4, 37, 38. Although a number of studies have indicated Ara h 2 to be the more potent of these two allergens 39–41, Ara h 1 also plays a major role in the pathogenesis of peanut allergy, with numerous studies reporting strong correlations between symptom severity and IgE reactivity to both Ara h 1 and Ara h 2 42–47. Ara h 1 is the most abundant major allergen in peanut, accounting for 12–16% of total peanut protein 48. This is an important consideration for driving linked epitope suppression in allergen immunotherapy, since inducing T cell suppressor activity against abundant major allergens will undoubtedly facilitate reduced responses to whole allergen extracts. We recently designed a panel of T cell epitope-based Ara h 2 peptides for inclusion in a peptide therapeutic 49. In a single report of sequences of T cell-reactive peptides from Ara h 1 using predictive tetramer-based epitope mapping 50, core epitopes were not determined and only 10 HLA-DR tetramers were used, preventing detection of epitopes presented on other HLA-types. Here, we provide a comprehensive report of precise core T cell epitopes of Ara h 1 based on analysis of full T cell repertoires from a large cohort of HLA-diverse peanut-allergic subjects. We reveal novel HLA-DQ-restricted epitopes as well as epitopes within previously reported T cell-reactive Ara h 1 20-mers 50. We also demonstrate presentation of the latter epitopes on additional HLA molecules to those previously reported 50. Using these sequences, we designed a panel of HLA-degenerate, T cell-reactive Ara h 1 peptides to combine with those we identified previously from Ara h 2 49, providing a broadly acting therapeutic to take forward for pre-clinical and clinical testing to treat HLA-diverse peanut-allergic populations.

## Methods

### Subjects

Peanut-allergic adult subjects were recruited from The Alfred Allergy Clinic, Melbourne, Australia (Table S1). All subjects had clinical symptoms of IgE-mediated peanut allergy and peanut-specific IgE CAP score ≥ 1 (≥ 0.49 kU_A_/l; Pharmacia CAP System™, Pharmacia Diagnostics, Uppsala, Sweden). Subjects used for T cell line (TCL) generation were genotyped (HLA-DRB1, -DQB1 and -DPB1, exon 2) by the Victorian Transplantation and Immunogenetics Service (Table S2). The study was approved by The Alfred and Monash University Ethics Committees and informed written consent was obtained from each subject.

### Antigens

Crude peanut extract (CPE) was prepared from commercial unsalted, dry-roasted peanuts as described 49, 51. Ara h 1 and Ara h 2 were enriched from CPE by liquid chromatography as described 49. Endotoxin contents were 1.7, 4.0 and 78.0 EU/mg for CPE, Ara h 1 and Ara h 2 respectively (Endpoint Chromogenic LAL assay; Lonza, Walkersville, MD, USA). Ara h 1 peptides (Mimotopes, Clayton, Victoria, Australia and GenScript USA Inc., Piscataway, New Jersey, USA; Table S3) were reconstituted at 2 mg/mL in 10% dimethyl sulfoxide/PBS (20-mers and truncated peptide sets) or PBS alone (custom-synthesized core epitope peptides). All antigens were confirmed to be neither mitogenic nor toxic as described 52.

### Generation of Ara h 1-specific CD4^+^ TCL

Ara h 1-specific oligoclonal TCL were generated from peripheral blood mononuclear cells (PBMC) of peanut-allergic subjects using 5,6-carboxyfluorescein diacetate succinimidylester (CFSE)-based methodology 53 as described 49, with CPE (100 μg/mL), Ara h 1 (10 μg/mL) or 20-mer peptides spanning the Ara h 1 sequence [11 amino acid (aa) overlap (17 aa overlap for the last peptide); Table S3; 10 μg/mL/peptide] as the driving antigens. All TCL were tested for specificity (proliferation) to individual Ara h 1 20-mers (10 μg/mL) as well as CPE (100 μg/mL) and/or Ara h 1 (10 μg/mL). Core epitope sequences were mapped within selected 20-mers using peptide sets truncated from the N- or C-terminus of the 20-mer as described 49.

### T cell assays

All culturing was performed in RPMI-1640 containing 2 mm l-glutamine, 100 IU/mL penicillin-streptomycin and 5% heat-inactivated human AB serum (Sigma-Aldrich, St Louis, MO, USA) (cRPMI). Antigen-induced TCL proliferation was assessed by ^3^H-thymidine (^3^H-TdR) uptake assays as described 49. A stimulation index (SI; cpm antigen-stimulated T cells/cpm unstimulated T cells) ≥ 2.5 was considered positive and all positive responses confirmed in ≥ 2 assays. HLA-restriction of epitope recognition by TCL was assessed using monoclonal antibodies (mAb) against HLA-DR (L243), HLA-DQ (SVP-L3) or HLA-DP (B7/21) to block epitope presentation as described 49. To allow detection of peptide-induced CD4^+^ T cell proliferation within whole PBMC, 7-day cultures of CFSE-labelled PBMC were set up as described for TCL generation 49. At least 10 000 CD4^+^ T cells were analysed per sample and SI calculated as percentage of CD4^+^CFSE^lo^ (proliferated) cells with antigen/percentage of CD4^+^CFSE^lo^ cells without antigen (background). The detection threshold for a specific response in this assay was assessed by expanding peptide-specific TCL from proliferated CD4^+^ cells over a range of SI values for three subjects. Specific TCL could be generated from divided T cells with SI as low as 1.1 in all three subjects (data not shown) allowing designation of an SI ≥ 1.1 as positive.

### Basophil activation test

Basophil activation was assessed by CD63 up-regulation detected by flow cytometry as described 54. Positive controls were rabbit anti-human IgE antibody (7.5 μg/mL; DAKO Corporation, Carpinteria, CA, USA), *N*-formyl-methionine-leucine-phenylalanine (fMLP) (0.4 μg/mL; Sigma) and CPE. CPE, Ara h 1 and peptides were tested over a 3-log concentration range (5, 0.5 and 0.05 μg/mL).

## Results

### Selection of Ara h 1 20-mer peptides containing CD4^+^ T cell epitopes most reliably recognized by peanut-allergic subjects

**Fig. 1:**
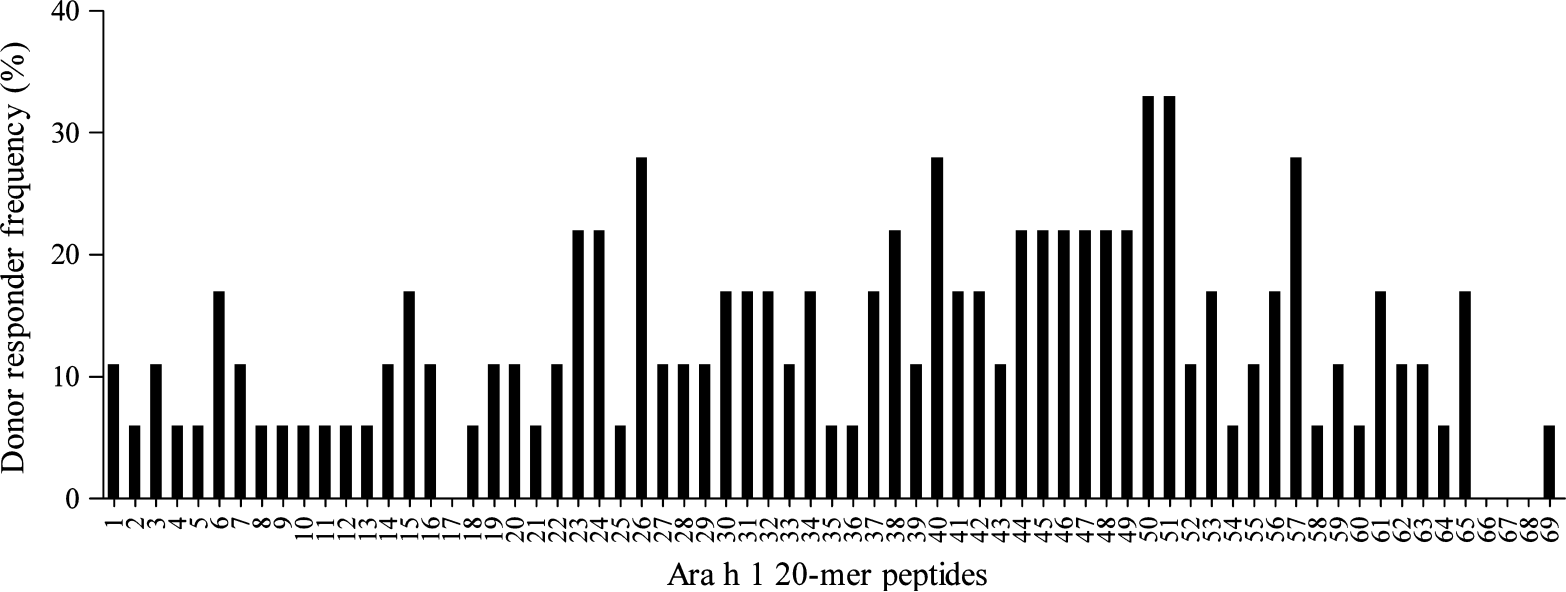
Donor responder frequency profile for Ara h 1 20-mer peptides. Donor responder frequencies for TCL recognition of Ara h 1 20-mer peptides (*n* = 18 peanut-allergic subjects).

A total of 145 Ara h 1-specific TCL were generated from PBMC of 18 HLA-diverse peanut-allergic subjects (Tables S1 and S2) by isolating and expanding antigen-specific (proliferated) CD4^+^CFSE^lo^ T cells from 7-day CFSE-labelled PBMC cultures stimulated with CPE, Ara h 1 or pools of Ara h 1 20-mer peptides collectively spanning the Ara h 1 sequence (Table S3). The 20-mer peptide(s) recognized (SI ≥ 2.5) by each subject are shown in Table 1 and data summarized in Fig. 1. For some subjects, CPE or Ara h 1 stimulation generated most TCL whilst for others it was the peptide pools. Where TCL were generated from a given subject using different antigen preparations (CPE, Ara h or peptide pools), TCL 20-mer specificities were comparable. Overall, there was no bias in the TCL 20-mer specificity generated depending on antigen preparation.

**Table 1:** Proliferative responses (thymidine uptake) of T cell lines to Ara h 1 20-mer peptides

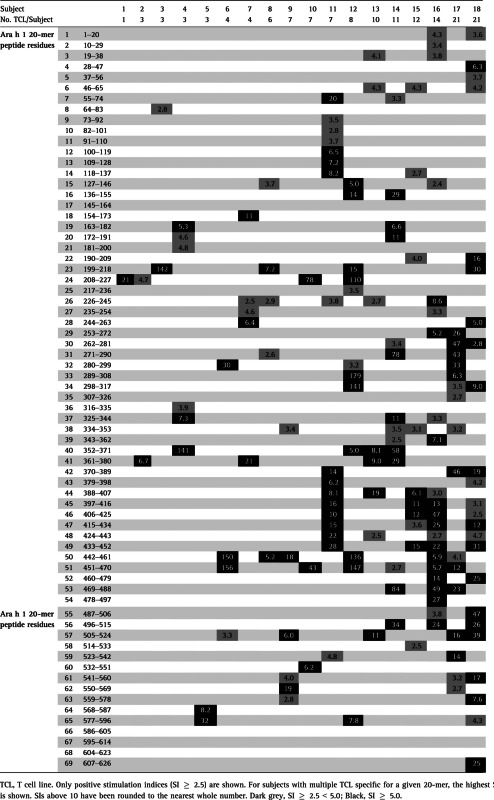

The 145 TCL collectively recognized epitopes throughout the entire Ara h 1 sequence, with only four of the sixty-nine 20-mers failing to stimulate any TCL. Fourteen 20-mers (23, 24, 26, 38, 40, 44–51 and 57) were each recognized by four (22%) or more subjects, with peptides 50 and 51 having the most responders (six subjects; 33%; Fig. 1). Although dominant allergen epitopes are most simply defined as being those peptides/regions most frequently recognized within the respective allergen sequence 55–59, we considered a number of factors in addition to TCL responder frequencies to further refine our selection of epitopes for inclusion in a therapeutic. These included the magnitude of TCL response, number of specific TCL per subject, reproducibility of specific TCL response and ability to target specific T cells in PBMC. Based on these parameters, nine of the fourteen 20-mers (peptides 23, 24, 40, 46, 47, 49, 50, 51 and 57) were selected for subsequent analyses. These nine 20-mers were collectively recognized by 16 of the 18 subjects (89%) in this cohort, and typically induced strong and consistent proliferative responses in specific TCL, with the majority of SI over 5 and many considerably higher (Table 1). Furthermore, each of these 20-mers was recognized by multiple TCL from many responders reflecting a prevalence of T cells specific for these peptides among the subjects’ T cell repertoires. To assess recognition in a wider cohort, PBMC from an additional 20 peanut-allergic subjects were screened by CFSE assay for CD4^+^ T cell proliferation in whole PBMC following 7 day stimulation with each peptide (Table 2, upper panel and Figure S1). This assay provided a sensitive and accurate screen for detecting the full repertoire of peptide-specific CD4^+^ T cell proliferative responses within whole PBMC. All 20 subjects showed PBMC T cell proliferation to CPE or a combination of enriched Ara h 1 and Ara h 2. The 20-mers were collectively recognized by 18 (90%) of these subjects, with 40–79% responding to each 20-mer. Analysis of four subjects from the original cohort used for TCL generation confirmed that they also had T cells specific for other 20-mers in addition to those recognized by their TCL (Table 2, lower panel). Combined totals for all 24 subjects tested with the CFSE assay showed 46–81% responded to each 20-mer. If a higher SI of 1.5 was used as a positive cut-off, the frequency of responders per 20-mer was only slightly reduced to 33–69%. Overall, T cell recognition of one or more of the selected panel of nine 20-mers was confirmed in 35 (92%) of 38 subjects analyzed using either cut-off.

**Table 2:** CFSE-based detection of peanut-allergic donor CD4^+^ T cell proliferation in response to selected Ara h 1 20-mers

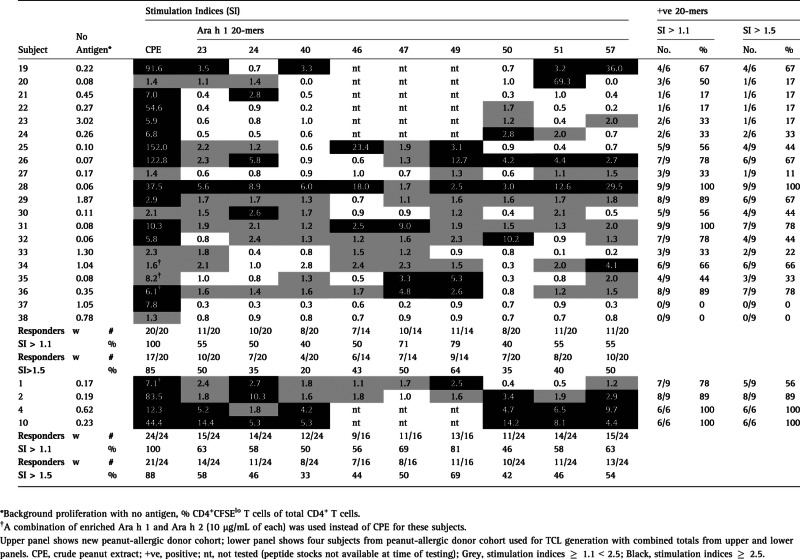

### Mapping core T cell epitopes within selected Ara h 1 20-mer peptides

**Fig. 2:**
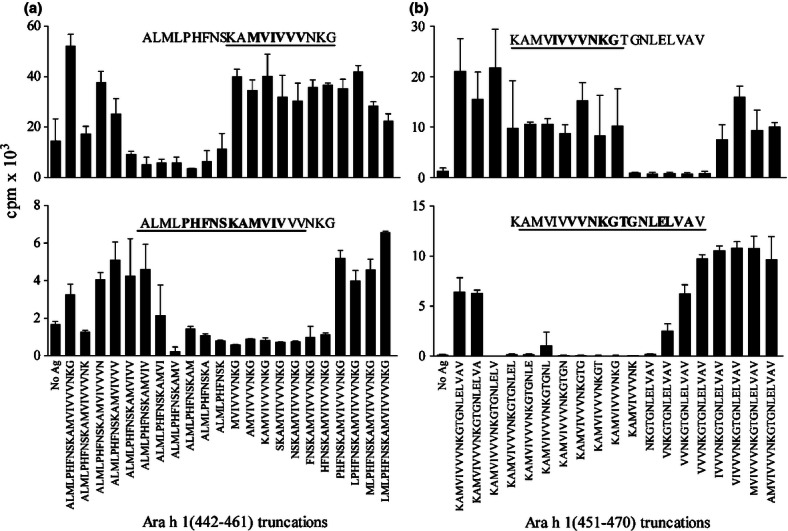
Mapping core T cell epitopes within Ara h 1 20-mer peptides 50 and 51. 20-mer-specific TCL proliferation to truncated peptide sets. Representative TCL shown for peptides 50 (a) and 51 (b) (mean cpm replicate wells + SD). Upper panels indicate the epitope in overlap between the 20-mers (*n* = 2; 3 TCL). Lower panels indicate epitopes unique to each 20-mer; (a) *n* = 3; 6 TCL. (b) *n* = 4; 7 TCL. Epitope sequences recognized by represented TCL are bolded and ‘consolidated epitopes’ recognized by all specific TCL are underlined.

Minimal length peptides decrease the risk of cross-linking cell-bound IgE on inflammatory cells during clinical administration and facilitate therapeutic production. The minimum T cell stimulatory sequence (core epitope) within each selected 20-mer was determined by testing the proliferation of reactive TCL from different subjects to truncated peptide sets (e.g. Fig. 2 and Table 3). The number of residues required to induce maximal T cell proliferation varied from 6 to 19 aa between different TCL and/or subjects (Table 3), consistent with previous reports for CD4^+^ T cell epitopes 60, 61. Due to variation in the number of flanking residues required for optimal epitope recognition 61, TCL were considered to recognize the same epitope if peptides containing a common core sequence induced recognition. Based on this criterion, 10 distinct CD4^+^ T cell epitopes were identified (‘consolidated epitopes’, Table 3), with common cores varying from 5 to 12 aa (underlined sequences, Table 3). ‘Consolidated epitope’ sequences were selected to encompass residues required for maximal stimulation of all specific TCL tested to ensure broadest possible recognition.

**Table 3:** Core T cell epitope sequences mapped within selected Ara h 1 20-mers

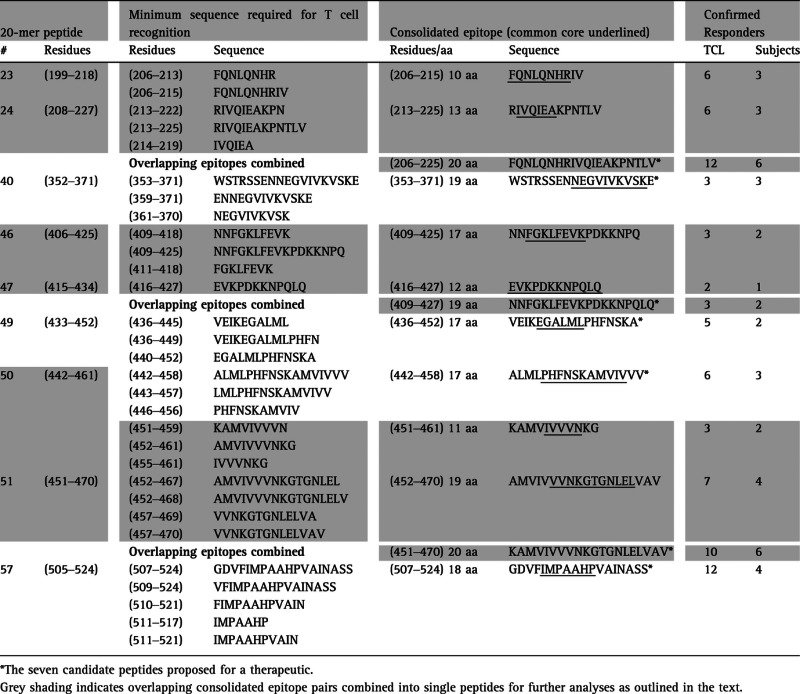

At least one epitope was found within each of the nine 20-mers, with 20-mers 50 and 51 each containing two distinct but overlapping T cell epitopes: one unique to each 20-mer (442–458 and 452–470), and the other within the overlap sequence (451–461, Table 3 and Fig. 2). No single TCL responded to both epitopes within either 20-mer, further confirming the distinction of these epitopes (data not shown). HLA-epitope prediction algorithms 62, 63 also highlighted one or more strong HLA class II (HLA-II) binding motifs within each of our minimal-stimulatory sequences. Data are shown for the Propred 62 HLA-DR binding algorithm in Table S4. This algorithm did not predict HLA-DR epitopes within peptide 40, but algorithms of the Immune Epitope Database (IEDB) and Analysis Resource 63 predicted epitopes within this peptide to bind most strongly to HLA-DP and/or -DQ molecules.

Finally, to avoid unnecessary sequence duplication and to minimize peptide numbers for a therapeutic, six of the consolidated epitopes (comprising three overlapping epitope pairs) were combined into three single peptides of 20 aa or less (206–225, 409–427 and 451–470; grey shading, Table 3). The combined epitope peptides efficiently stimulated TCL specific for either epitope (data not shown) and together with the remaining four consolidated epitopes (353–371, 436–452, 442–458 and 507–524), provided a panel of seven candidate peptides for further characterization (see asterisks, Table 3). CFSE-based screening of nine subjects from our cohorts confirmed that these peptides could each directly target detectable numbers of Ara h 1-specific T cells among whole PBMC of peanut-allergic subjects (Table 4). In a few cases, the T cell response of a given subject to the original 20-mer and the corresponding candidate peptide differed. Where responses to candidates were reduced as compared to the 20-mer, flanking residue(s) required for optimal T cell recognition or HLA-binding may have been removed. It is well recognized that flanking residues can stabilize peptide binding to HLA class II molecules. In contrast, improved responses could reflect generation of additional new epitopes, improved epitope purity [cores were synthesized at high purity (> 95%), whilst 20-mers were produced as peptide sets with a minimum estimated purity of 70%] or alteration of epitopes (or flanking residues) to enable better interaction with HLA and/or T cell receptor (TCR) molecules. Indeed, this was considered the case where responses to candidate peptide (442–458) were much stronger than to 20-mer 50. As this region contains multiple adjacent hydrophobic residues, even single residue changes could significantly alter the charge and structure of this peptide, thus affecting its biochemical properties and interactions with HLA and/or TCR molecules. Nonetheless, most responses to candidates were comparable or improved compared to responses to the original 20-mers.

**Table 4:** CFSE-based detection of peanut-allergic donor CD4+ T cell proliferation in response to selected Ara h 1 candidate peptides

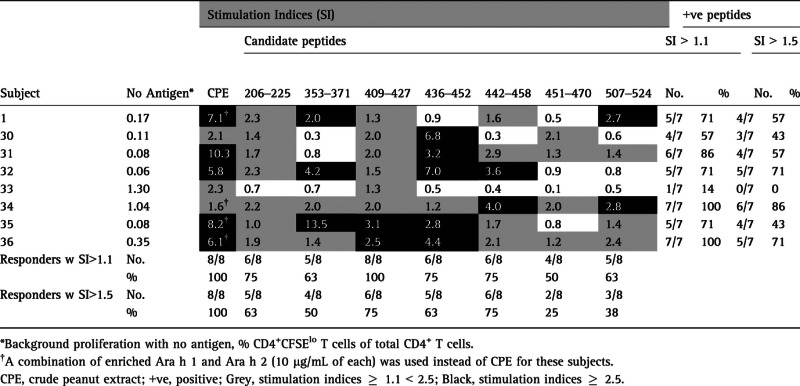

### Determining HLA class II restriction specificity of Ara h 1 T cell epitopes

There is no identified HLA-II association with peanut allergy 64, therefore peptides selected for therapy must bind diverse HLA-II molecules for wide applicability. To determine the HLA-II type presenting each epitope, anti-HLA-DR, -DP or -DQ mAbs were used to block individual epitope presentation to T cells. For each TCL tested, epitope recognition was prevented by one or more HLA-mAb in a dose-dependent manner (e.g. Figure S2) and the same mAb blocked recognition of CPE (data not shown), demonstrating consistency for presentation of naturally processed and synthetic epitope forms. At least two subjects and/or TCL were tested per epitope (Table 5). Consistent with predictions of the HLA-II algorithms described above 62, 63, anti-HLA-DR blocked recognition of all but one epitope (353–371), which was blocked by anti-HLA-DQ in both subjects tested. For epitopes 436–452 and 507–524, recognition was blocked by anti-HLA-DR for some TCL but by anti-HLA-DQ for others, confirming HLA-binding degeneracy for these epitopes.

**Table 5:** HLA class II restriction of core epitope peptides

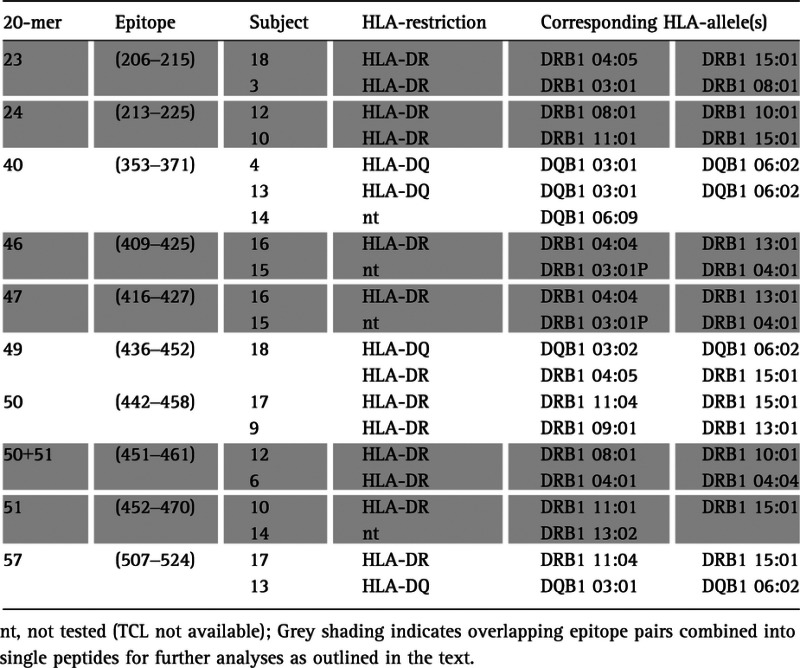

To assess HLA-binding degeneracy of epitopes whose recognition was blocked by a single HLA-mAb, the respective HLA-alleles of at least two subjects with TCL specific for that epitope were compared (Table S2 and Table 5). The absence of shared *HLA-DRB1* or *HLA-DQB1* alleles between subjects recognizing HLA-DR- or HLA-DQ-restricted epitopes, respectively, confirmed that each epitope was present on at least two different HLA-molecules. The HLA-binding algorithms further supported these data, with each epitope containing motifs predicted to bind multiple HLA molecules 62, 63 (e.g. Table S4).

### Testing candidate peptides for basophil activation

**Fig. 3:**
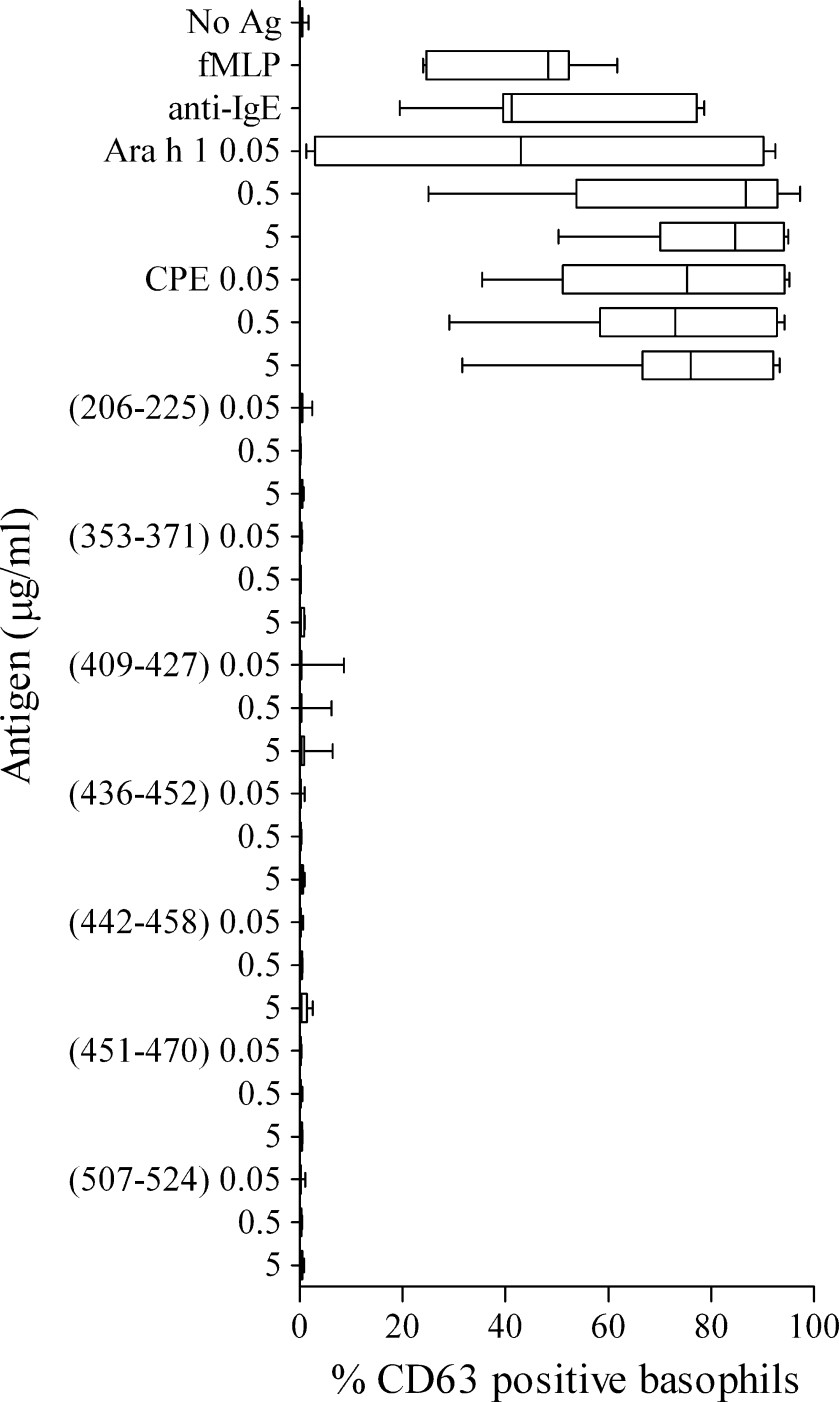
Basophil activation in response to candidate Ara h 1 peptides. Box-and-whiskers plot showing percentage of activated (CD63^hi^) basophils (IgE^hi^) in response to Ara h 1 or candidate peptides for seven peanut-allergic subjects. Negative control was no antigen (unstimulated) and positive controls were anti-IgE, fMLP and CPE. Whiskers show minimum to maximum values.

To provide a safe alternative to whole allergens, peptides must not bind and cross-link cell-bound IgE. Basophil reactivity to peptides was assessed in fresh blood from seven of the peanut-allergic subjects recruited for this study (Fig. 3). All seven subjects showed high levels of basophil activation to CPE over a concentration range. Whilst responses to Ara h 1 varied between subjects at the lowest dose, the highest concentration induced high activation in all subjects. However, none of the candidate peptides induced activation at any concentration tested. One subject showed a very low response (8%) to peptide 409–427, but this was below the threshold of positive activation 65 and was negligible compared to the activation induced by Ara h 1 (80–90%) or CPE (74–76%) in this subject.

## Discussion

An appropriately selected T cell-targeted peptide immunotherapeutic will provide a safe treatment option for peanut-allergic individuals. Candidate peptides must comprise HLA-degenerate CD4^+^ T cell epitopes of the major peanut allergens recognized by HLA-diverse peanut-allergic individuals, without cross-linking cell-bound IgE and activating inflammatory cells. To maximize the population coverage and efficacy of a therapeutic, we designed a peptide set containing T cell epitopes from the two major allergens Ara h 1 and Ara h 2. Following on from our previous study of Ara h 2 49, we now provide the first report of core sequences of CD4^+^ T cell epitopes of the most abundant major peanut allergen Ara h 1. We identified and characterized 10 HLA-diverse CD4^+^ T cell epitopes of Ara h 1, and used these sequences to design candidate Ara h 1 peptides to combine with our candidate Ara h 2 peptides for therapeutic development.

To commence this study, we selected nine 20-mers of Ara h 1 as containing the most frequently recognized epitopes based on responses of 145 TCL from 18 HLA-diverse peanut-allergic subjects. The cohort HLA profile was typical of Caucasian populations 66 in countries where peanut allergy is prevalent 67. We further validated our 20-mer selection by demonstrating their collective recognition by PBMC T cells directly *ex vivo* from an additional 18 peanut-allergic subjects (Table 2), resulting in a total responder frequency of 92% for the 38 subjects analysed. Although we could not confirm T cell recognition of these 20-mers in four subjects, it is possible that specific T cells went undetected for two of these subjects (5 and 7), as data were only obtained from three and four TCL respectively (Table 1).

The minimum T cell stimulatory sequences identified within our selected 20-mers varied from 6 to 19 aa (Table 3), consistent with reports of different peptides processed for HLA-II presentation and/or required for HLA- and/or TCR-binding both within and between subjects 60, 61. As we used oligoclonal TCL, it is possible that the longer sequences contained more than one epitope. Indeed, algorithms 62, 63 predicted up to three HLA-binding motifs within some of our consolidated epitopes (Table S4). Seven of our epitopes showed overlap with T cell-reactive Ara h 1 20-mers recently identified using HLA-DR tetramers 50, providing further support for recognition of these peptides in larger peanut-allergic populations. In addition, we confirmed the presentation of five of these peptides on additional HLA molecules to those used for the tetramer mapping 50. However, epitopes 353–371, 436–452 and 442–458 were unique to our study. Consistent with this observation, we showed these epitopes were either presented on HLA-DQ molecules (commonly observed for allergen T cell epitopes 68–72), or HLA-DR types for which no tetramer-specific T cells were detected 50 (Table 5). Inclusion of HLA-DQ-restricted epitopes is particularly advantageous for therapeutics as these alleles are less variable and thus more prevalent in mixed populations than HLA-DR alleles 73. However, we confirmed HLA-binding degeneracy for all epitopes identified (both HLA-DR and -DQ-restricted) (Table 5), with further degeneracy predicted by algorithms 62, 63 (e.g. Table S4), emphasizing their collective suitability for targeting HLA-diverse peanut-allergic populations.

The main rationale for developing a peptide immunotherapy for peanut allergy is to identify an effective allergen preparation that does not invoke the adverse effects seen with whole peanut extract 10, 12–14, 19, 37, 74. Mapping the core sequences of T cell epitopes enables refined peptide design for a therapeutic, but selecting optimal peptide combinations is a balance between peptide length and number. Longer peptides will increase population coverage by encompassing more T cell epitopes, and being fewer in number will reduce the complexity of therapeutic standardization compared to using a greater number of shorter peptides. However, the main concern with longer peptides is the increased potential for IgE binding and cross-linking, resulting in adverse reactions. We opted to combine overlapping epitopes into peptides up to 20 aa in length. Of over 23 linear IgE epitopes reported for Ara h 1 75–77, only two minor epitopes (409–418 and 461–470) fell within our candidate peptides 75. Most importantly, none of these peptides caused activation of peanut-reactive basophils in all of the seven peanut-allergic subjects tested (Fig. 3), emphasizing the potential for these peptides to provide a safe alternative to whole allergen extract for immunotherapy.

In summary, we report the novel identification of 10 reliably recognized CD4^+^ T cell epitopes of Ara h 1 that collectively show diverse HLA class II restriction. We incorporated these epitopes into a panel of seven short (≤ 20 aa), HLA-degenerate peptides that can target T cells within PBMC of HLA-diverse allergic individuals without causing activation of peanut-allergic donor basophils. The combination of these Ara h 1 peptides with our three T cell epitope-based Ara h 2 peptides 49 provides strong candidates for a broad acting and safe peptide-therapeutic to treat peanut allergy.

## Supporting Information

Figure S1. Representative CFSE-based assay for detecting CD4+ T cell proliferation in PBMC.

Figure S2. Representative HLA class II restriction specificity of T cell epitope recognition.

Table S1. Subject demographics.

Table S2. HLA genotyping for subjects used for T cell line generation.

Table S3. Ara h 1 20-mer peptides.

Table S4. Predicted HLA-DR binding motifs in selected Ara h 1 20-mers.
