# Supplementary material for: Ara h 1 CD4+ T cell epitope-based peptides: candidates for a peanut allergy therapeutic
Source: Clin Exp Allergy. 2013 May 28;43(6):684–97. doi: 10.1111/cea.12113 (PMC3709139; doi:10.1111/cea.12113)
Supplement: Supplementary file 3 [file cea0043-0684-SD3.docx]

**Online Supporting Information**

Original Article

**Ara h 1 CD4+ T-cell epitope-based peptides: candidates for a peanut allergy therapeutic**

Sara R Prickett ^1,2^, Astrid L Voskamp ^1,2^, Tracy Phan ^1,2^, April Dacumos-Hill ^1,2^, Stuart I Mannering ^3^, Jennifer M Rolland ^1,2^, and Robyn E O’Hehir ^1,2^

^1^Department of Immunology, Monash University, Melbourne, Victoria, Australia

^2^Department of Allergy, Immunology and Respiratory Medicine, The Alfred Hospital and Monash University, Melbourne, Victoria, Australia

^3^Immunology and Diabetes, St Vincent’s Institute of Medical Research, Melbourne, Victoria, Australia

**Corresponding Author:**

Name: Professor Robyn O’Hehir

Address: Department of Allergy, Immunology and Respiratory Medicine, The Alfred Hospital and Monash University, Commercial Road, Melbourne 3004, Victoria, Australia.

Email: r.ohehir@alfred.org.au

Phone: +61 3 9076 2251

Fax: +61 3 9076 8692

**Table S1.** Subject demographics

| **Sub-ject** | **Sex** | **Age** | **Atopic*** | **Asthma** | **Peanut CAP**  **kU_A_/l (score)** | **Anaph-ylaxis** | **Use of patient samples** | | | |
| --- | --- | --- | --- | --- | --- | --- | --- | --- | --- | --- |
|  |  |  |  |  |  |  | **TCL** | **20-mer CFSE** | **Core CFSE** | **BAT** |
|  |  |  |  |  |  |  |  |  |  |  |
| 1 | M | 39 | Yes | No | 2.18 (2) | Yes | X | X | X |  |
| 2 | M | 34 | Yes | Yes | 0.78 (2) | Yes | X | X |  |  |
| 3 | F | 53 | Yes | as a child | 83.90 (5) | Yes | X |  |  |  |
| 4 | F | 19 | Yes | No | 98.90 (5) | Yes | X | X |  |  |
| 5 | F | 22 | Yes | No | 4.72 (3) | Yes | X |  |  |  |
| 6 | M | 30 | Yes | No | 17.00 (4) | Yes | X |  |  |  |
| 7 | M | 42 | No | No | 15.40 (3) | Yes | X |  |  |  |
| 8 | M | 36 | Yes | Yes | 56.60 (5) | Yes | X |  |  |  |
| 9 | M | 30 | Yes | Yes | 30.60 (4) | Yes | X |  |  |  |
| 10 | M | 37 | Yes | Yes | 42.70 (4) | Yes | X | X |  | X |
| 11 | F | 26 | Yes | Yes | 2.82 (2) | Yes | X |  |  |  |
| 12 | F | 23 | Yes | Yes | >100 (6) | Yes | X |  |  |  |
| 13 | M | 30 | Yes | No | >100 (6) | Yes | X |  |  |  |
| 14 | M | 30 | Yes | Yes | 36.60 (4) | Yes | X |  |  | X |
| 15 | F | 31 | Yes | No | 84.30 (5) | No | X |  |  |  |
| 16 | F | 20 | Yes | Yes | 1.16 (2) | Yes | X |  |  |  |
| 17 | F | 25 | Yes | No | 2.12 (2) | Yes | X |  |  | X |
| 18 | M | 35 | Yes | Yes | 1.23 (2) | No | X |  |  |  |
| 19 | M | 27 | Yes | Yes | 6.19 (3) | Yes |  | X |  |  |
| 20 | F | 25 | Yes | Yes | 87.2 (5) | Yes |  | X |  |  |
| 21 | F | 53 | Yes | No | 1.43 (2) | No |  | X |  |  |
| 22 | F | 28 | Yes | Yes | 9.53 (3) | na |  | X |  |  |
| 23 | F | 37 | Yes | No | 6.94 (3) | Yes |  | X |  |  |
| 24 | M | 38 | Yes | Yes | 2.42 (2) | Yes |  | X |  |  |
| 25 | M | 28 | Yes | Yes | >100 (6) | Yes |  | X |  |  |
| 26 | F | 70 | No | No | 2.18 (2) | Yes |  | X |  | X |
| 27 | F | 26 | Yes | No | 1.37 (2) | No |  | X |  |  |
| 28 | F | 35 | Yes | No | SPT 14mm | Yes |  | X |  |  |
| 29 | F | 23 | na | No | 2.37 (2) | na |  | X |  |  |
| 30 | F | 28 | Yes | Yes | 9.2 (3) | No |  | X | X |  |
| 31 | F | 30 | Yes | Yes | 10.20 (3) | Yes |  | X | X |  |
| 32 | M | 53 | Yes | No | 2.01 (2) | Yes |  | X | X |  |
| 33 | M | 26 | Yes | Yes | 12.00(3) | Yes |  | X | X |  |
| 34 | M | 43 | Yes | Yes | 1.63 (2) | No |  | X | X | X |
| 35 | F | 33 | Yes | na | 0.49 (1) | No |  | X | X |  |
| 36 | M | 28 | Yes | na | 0.72 (2) | no |  | X | X |  |
| 37 | F | 21 | Yes | Yes | 1.51 (2) | Yes |  | X |  |  |
| 38 | M | 28 | Yes | Yes | 1.43(2) | Yes |  | X |  |  |
| 39 | M | 29 | Yes | No | 31.80 (4) | Yes |  |  |  | X |
| 40 | F | 52 | Yes | Yes | 7.23 (3) | Yes |  |  |  | X |

* Atopic is defined by specific IgE to one or more of a panel of common aeroallergens either by RAST or skin prick test.

TCL, T cell line; 20-mer CFSE, screen for T cell reactivity to selected Ara h 1 20-mers; Core CFSE, screen for T cell reactivity to candidate Ara h 1 peptides; BAT, basophil activation test; na, data not available; SPT, skin-prick test (RAST not available for this subject).

**Table S2.** HLA genotyping for subjects used for T-cell line generation

| **Subject** | **HLA-genotypes** | | | | | | | | | | |
| --- | --- | --- | --- | --- | --- | --- | --- | --- | --- | --- | --- |
|  | **DRB1** | | | | **DQB1** | | | | **DPB1** | | |
|  |  |  | |  | |  | |  | |  | |
| 1 | 07:01 | | 15:01 | | 02:01 | | 06:02 | | 04:01 | |  |
| 2 | 01:01 | | 03:01 | | 05:01 | | 06:02 | | 04:01 | | 04:02 |
| 3 | 03:01 | | 08:01 | | 02:01P | | 04:02 | | 03:01P | | 04:01 |
| 4 | 11:01 | | 15:01 | | 03:01P | | 06:02 | | 04:01 | |  |
| 5 | 11:01 | | 15:01 | | 03:01P | | 06:02 | | 03:01P | | 04:01 |
| 6 | 04:01 | | 04:04 | | 03:02 | | 04:02 | | 13:01P | | 04:01 |
| 7 | 07:01 | | 08:01 | | 03:03 | | 04:02 | | 04:01 | | 06:01 |
| 8 | 01:03 | | 04:01 | | 03:02 | | 05:01 | | 03:01P | | 02:01 |
| 9 | 09:01 | | 13:01 | | 03:03 | | 06:03 | | 03:01P | | 04:02P |
| 10 | 11:01 | | 15:01 | | 03:01P | | 06:02 | | 04:01 | |  |
| 11 | 03:01 | | 13:02 | | 02:01P | | 06:09 | | 01:01 | | 04:01 |
| 12 | 08:01 | | 10:01 | | 04:02 | | 05:01 | | 03:01P | | 04:01 |
| 13 | 12:01P | | 15:01 | | 03:01 | | 06:02 | | 13:01P | | 04:01 |
| 14 | 13:02 | |  | | 06:09 | |  | | 05:01 | | 04:02P |
| 15 | 03:01P | | 04:01 | | 04:01P | |  | | 02:01P | | 03:01P |
| 16 | 04:04 | | 13:01 | | 03:02 | | 06:03 | | 02:01 | | 04:01 |
| 17 | 11:04 | | 15:01 | | 03:01P | | 06:02 | | 02:01 | | 14:01 |
| 18 | 04:05 | | 15:01 | | 03:02 | | 06:02 | | 03:01P | | 04:01 |

All HLA abbreviations comply with recent changes to allele nomenclature (http://hla.alleles.org/announcement.html and http://www.ebi.ac.uk/imgt/hla/).

Alleles followed by a ‘P’ represent groups of alleles that share common sequences in exon 2 (http://hla.alleles.org/alleles/p_groups.html).

**Table S3.** Ara h 1 20-mer peptides

| **Pool** | **No.** | **Residues** | **Sequence** |  |  | **Pool** | **No.** | **Residues** | **Sequence** |
| --- | --- | --- | --- | --- | --- | --- | --- | --- | --- |
|  |  |  |  |  |  |  |  |  |  |
| 1 | 1 | 1-20 | MRGRVSPLMLLLGILVLASV |  |  | 6 | 36 | 316-335 | FSRNTLEAAFNAEFNEIRRV |
|  | 2 | 10-29 | LLLGILVLASVSATHAKSSP |  |  |  | 37 | 325-344 | FNAEFNEIRRVLLEENAGGE |
|  | 3 | 19-38 | SVSATHAKSSPYQKKTENPC |  |  |  | 38 | 334-353 | RVLLEENAGGEQEERGQRRW |
|  | 4 | 28-47 | SPYQKKTENPCAQRCLQSCQ |  |  |  | 39 | 343-362 | GEQEERGQRRWSTRSSENNE |
|  | 5 | 37-56 | PCAQRCLQSCQQEPDDLKQK |  |  |  | 40 | 352-371 | RWSTRSSENNEGVIVKVSKE |
|  | 6 | 46-65 | CQQEPDDLKQKACESRCTKL |  |  |  | 41 | 361-380 | NEGVIVKVSKEHVEELTKHA |
|  | 7 | 55-74 | QKACESRCTKLEYDPRCVYD |  |  |  | 42 | 370-389 | KEHVEELTKHAKSVSKKGSE |
|  |  |  |  |  |  |  |  |  |  |
| 2 | 8 | 64-83 | KLEYDPRCVYDPRGHTGTTN |  |  | 7 | 43 | 379-398 | HAKSVSKKGSEEEGDITNPI |
|  | 9 | 73-92 | YDPRGHTGTTNQRSPPGERT |  |  |  | 44 | 388-407 | SEEEGDITNPINLREGEPDL |
|  | 10 | 82-101 | TNQRSPPGERTRGRQPGDYD |  |  |  | 45 | 397-416 | PINLREGEPDLSNNFGKLFE |
|  | 11 | 91-110 | RTRGRQPGDYDDDRRQPRRE |  |  |  | 46 | 406-425 | DLSNNFGKLFEVKPDKKNPQ |
|  | 12 | 100-119 | YDDDRRQPRREEGGRWGPAG |  |  |  | 47 | 415-434 | FEVKPDKKNPQLQDLDMMLT |
|  | 13 | 109-128 | REEGGRWGPAGPREREREED |  |  |  | 48 | 424-443 | PQLQDLDMMLTCVEIKEGAL |
|  | 14 | 118-137 | AGPREREREEDWRQPREDWR |  |  |  | 49 | 433-452 | LTCVEIKEGALMLPHFNSKA |
|  |  |  |  |  |  |  |  |  |  |
| 3 | 15 | 127-146 | EDWRQPREDWRRPSHQQPRK |  |  | 8 | 50 | 442-461 | ALMLPHFNSKAMVIVVVNKG |
|  | 16 | 136-155 | WRRPSHQQPRKIRPEGREGE |  |  |  | 51 | 451-470 | KAMVIVVVNKGTGNLELVAV |
|  | 17 | 145-164 | RKIRPEGREGEQEWGTPGSH |  |  |  | 52 | 460-479 | KGTGNLELVAVRKEQQQRGR |
|  | 18 | 154-173 | GEQEWGTPGSHVREETSRNN |  |  |  | 53 | 469-488 | AVRKEQQQRGRREEEEDEDE |
|  | 19 | 163-182 | SHVREETSRNNPFYFPSRRF |  |  |  | 54 | 478-497 | GRREEEEDEDEEEEGSNREV |
|  | 20 | 172-191 | NNPFYFPSRRFSTRYGNQNG |  |  |  | 55 | 487-506 | DEEEEGSNREVRRYTARLKE |
|  | 21 | 181-200 | RFSTRYGNQNGRIRVLQRFD |  |  |  | 56 | 496-515 | EVRRYTARLKEGDVFIMPAA |
|  |  |  |  |  |  |  |  |  |  |
| 4 | 22 | 190-209 | NGRIRVLQRFDQRSRQFQNL |  |  | 9 | 57 | 505-524 | KEGDVFIMPAAHPVAINASS |
|  | 23 | 199-218 | FDQRSRQFQNLQNHRIVQIE |  |  |  | 58 | 514-533 | AAHPVAINASSELHLLGFGI |
|  | 24 | 208-227 | NLQNHRIVQIEAKPNTLVLP |  |  |  | 59 | 523-542 | SSELHLLGFGINAENNHRIF |
|  | 25 | 217-236 | IEAKPNTLVLPKHADADNIL |  |  |  | 60 | 532-551 | GINAENNHRIFLAGDKDNVI |
|  | 26 | 226-245 | LPKHADADNILVIQQGQATV |  |  |  | 61 | 541-560 | IFLAGDKDNVIDQIEKQAKD |
|  | 27 | 235-254 | ILVIQQGQATVTVANGNNRK |  |  |  | 62 | 550-569 | VIDQIEKQAKDLAFPGSGEQ |
|  | 28 | 244-263 | TVTVANGNNRKSFNLDEGHA |  |  |  | 63 | 559-578 | KDLAFPGSGEQVEKLIKNQK |
|  |  |  |  |  |  |  |  |  |  |
| 5 | 29 | 253-272 | RKSFNLDEGHALRIPSGFIS |  |  | 10 | 64 | 568-587 | EQVEKLIKNQKESHFVSARP |
|  | 30 | 262-281 | HALRIPSGFISYILNRHDNQ |  |  |  | 65 | 577-596 | QKESHFVSARPQSQSQSPSS |
|  | 31 | 271-290 | ISYILNRHDNQNLRVAKISM |  |  |  | 66 | 586-605 | RPQSQSQSPSSPEKESPEKE |
|  | 32 | 280-299 | NQNLRVAKISMPVNTPGQFE |  |  |  | 67 | 595-614 | SSPEKESPEKEDQEEENQGG |
|  | 33 | 289-308 | SMPVNTPGQFEDFFPASSRD |  |  |  | 68 | 604-623 | KEDQEEENQGGKGPLLSILK |
|  | 34 | 298-317 | FEDFFPASSRDQSSYLQGFS |  |  |  | 69 | 607-626 | QEEENQGGKGPLLSILKAFN |
|  | 35 | 307-326 | RDQSSYLQGFSRNTLEAAFN |  |  |  |  |  |  |

**Table S4.** Predicted HLA-DR binding motifs in selected Ara h 1 20-mers

| **HLA molecule** | **Ara h 1 20-mer peptide** | | | | | | | | |
| --- | --- | --- | --- | --- | --- | --- | --- | --- | --- |
|  | **23 (199-218)** | **24 (208-227)** | **46 (406-425)** | **47 (415-434)** | **49 (433-452)** | **50 (442-461)** | **51 (451-470)** | **57 (505-524)** |  |
| DRB1_0101 | FDQRSRQ**F**QNLQNHRIVQIE | NLQNHR**I**VQIEAKPNTLVLP | DLSNNFGKLFEVKPDKKNPQ | FEVKPDKKNPQLQDLDMMLT | LTCVEIKEGALMLPHFNSKA | ALMLPHFNSKA**M**VIVVVNKG | KA**M**VI**V**VVNKGTGNLELVAV | KEGD**VFI**MPAAHPVAINASS |  |
| DRB1_0102 | FDQRSRQ**F**QNLQNHRIVQIE | NLQNHR**I**VQIEAKPNTLVLP | DLSNNFGKLFEVKPDKKNPQ | FEVKPDKKNPQLQDLDMMLT | LTCVEIKEGALMLPHFNSKA | ALMLPHFNSKA**M**VIVVVNKG | KA**M**VI**V**VVNKGTGNLELVAV | KEGD**VF**I**M**PAAHPVAINASS |  |
| DRB1_0301 | FDQRSRQFQN**L**QNHRIVQIE | N**L**QNHRI**V**QIEAKPNTLVLP | DLSNNFGKLFE**V**KPDKKNPQ | FE**V**KPDKKNPQLQDLDMMLT | LTCVEIKEGALMLPHFNSKA | ALMLPHFNSKA**M**VIVVVNKG | KA**M**V**I**VVVNKGTGNLELVAV | KEGDVFIMPAAHPVAINASS |  |
| DRB1_0305 | FDQRSRQFQNLQNHRIVQIE | NLQNHRIVQIEAKPNTLVLP | DLSNNFGKLFE**V**KPDKKNPQ | FE**V**KPDKKNPQLQDLDMMLT | LTCVEIKEGALMLPHFNSKA | ALMLPHFNSKA**M**VIVVVNKG | KA**M**V**I**VVVNKGTGNLELVAV | KEGDVFIMPAAHPVAINASS |  |
| DRB1_0306 | FDQRSRQFQN**L**QNHRIVQIE | N**L**QNHRIVQIEAKPNTLVLP | DLSNNFGKLFE**V**KPDKKNPQ | FE**V**KPDKKNPQLQDLDMMLT | LTCVEIKEGALMLPHFNSKA | ALMLPHFNSKA**M**VIVVVNKG | KA**M**VIVVVNKGTGNLELVAV | KEGDVFIMPAAHPVAINASS |  |
| DRB1_0307 | FDQRSRQFQN**L**QNHRIVQIE | N**L**QNHRIVQIEAKPNTLVLP | DLSNNFGKLFE**V**KPDKKNPQ | FE**V**KPDKKNPQLQDLDMMLT | LTCVEIKEGALMLPHFNSKA | ALMLPHFNSKA**M**VIVVVNKG | KA**M**VIVVVNKGTGNLELVAV | KEGDVFIMPAAHPVAINASS |  |
| DRB1_0308 | FDQRSRQFQN**L**QNHRIVQIE | N**L**QNHRIVQIEAKPNTLVLP | DLSNNFGKLFE**V**KPDKKNPQ | FE**V**KPDKKNPQLQDLDMMLT | LTCVEIKEGALMLPHFNSKA | ALMLPHFNSKA**M**VIVVVNKG | KA**M**VIVVVNKGTGNLELVAV | KEGDVFIMPAAHPVAINASS |  |
| DRB1_0309 | FDQRSRQFQNLQNHRIVQIE | NLQNHRIVQIEAKPNTLVLP | DLSNNFGKLFE**V**KPDKKNPQ | FE**V**KPDKKNPQLQDLDMMLT | LTCVEIKEGALMLPHFNSKA | ALMLPHFNSKA**M**VIVVVNKG | KA**M**V**I**VVVNKGTGNLELVAV | KEGDVFIMPAAHPVAINASS |  |
| DRB1_0311 | FDQRSRQFQN**L**QNHRIVQIE | N**L**QNHRIVQIEAKPNTLVLP | DLSNNFGKLFE**V**KPDKKNPQ | FE**V**KPDKKNPQLQDLDMMLT | LTCVEIKEGALMLPHFNSKA | ALMLPHFNSKA**M**VIVVVNKG | KA**M**VIVVVNKGTGNLELVAV | KEGDVFIMPAAHPVAINASS |  |
| DRB1_0401 | FDQRSRQ**F**QNLQNHRIVQIE | N**L**QNHRIVQIEAKPNTLVLP | DLSNNFGKLFEVKPDKKNPQ | FEVKPDKKNPQLQDLDMMLT | LTCVEIKEGAL**M**LPHFNSKA | AL**M**LPHFNSKA**M**VIVVVNKG | KA**M**VIVVVNKGTGNLELVAV | KEGDVFIMPAAHPVAINASS |  |
| DRB1_0402 | FDQRSRQFQNLQNHRIVQIE | N**L**QNHRIVQIEAKPNTLVLP | DLSNNFGKLFEVKPDKKNPQ | FEVKPDKKNPQLQDLDMMLT | LTCVEIKEGAL**M**LPHFNSKA | AL**M**LPHFNSKA**M**VIVVVNKG | KA**M**VIVVVNKGTGNLELVAV | KEGDVFIMPAAHPVAINASS |  |
| DRB1_0404 | FDQRSRQ**F**QNLQNHRIVQIE | NLQNHRIVQIEAKPNTLVLP | DLSNNFGKLFEVKPDKKNPQ | FEVKPDKKNPQLQDLDMMLT | LTCVEIKEGAL**M**LPHFNSKA | AL**M**LPHFNSKA**M**VIVVVNKG | KA**M**VIVVVNKGTGNLELVAV | KEGD**V**FIMPAAHPVAINASS |  |
| DRB1_0405 | FDQRSRQ**F**QNLQNHRIVQIE | NLQNHRIVQIEAKPNTLVLP | DLSNNFGKLFEVKPDKKNPQ | FEVKPDKKNPQLQDLDMMLT | LTCVEIKEGALMLPHFNSKA | ALMLPHFNSKA**M**VIVVVNKG | KA**M**VIVVVNKGTGNLELVAV | KEGD**V**FIMPAAHPVAINASS |  |
| DRB1_0408 | FDQRSRQ**F**QNLQNHRIVQIE | NLQNHRIVQIEAKPNTLVLP | DLSNNFGKLFEVKPDKKNPQ | FEVKPDKKNPQLQDLDMMLT | LTCVEIKEGALMLPHFNSKA | ALMLPHFNSKA**M**VIVVVNKG | KA**M**VIVVVNKGTGNLELVAV | KEGD**V**FIMPAAHPVAINASS |  |
| DRB1_0410 | FDQRSRQFQNLQNHRIVQIE | NLQNHRIVQIEAKPNTLVLP | DLSNNFGKLFEVKPDKKNPQ | FEVKPDKKNPQLQDLDMMLT | LTCVEIKEGALMLPHFNSKA | ALMLPHFNSKA**M**VIVVVNKG | KA**M**VIVVVNKGTGNLELVAV | KEGD**V**FIMPAAHPVAINASS |  |
| DRB1_0421 | FDQRSRQ**F**QN**L**QNHRIVQIE | N**L**QNHRIVQIEAKPNTLVLP | DLSNNFGKLFEVKPDKKNPQ | FEVKPDKKNPQLQDLDMMLT | LTCVEIKEGAL**M**LPHFNSKA | AL**M**LPHFNSKA**M**VIVVVNKG | KA**M**VIVVVNKGTGNLELVAV | KEGDVFIMPAAHPVAINASS |  |
| DRB1_0423 | FDQRSRQ**F**QNLQNHRIVQIE | NLQNHRIVQIEAKPNTLVLP | DLSNNFGKLFEVKPDKKNPQ | FEVKPDKKNPQLQDLDMMLT | LTCVEIKEGAL**M**LPHFNSKA | AL**M**LPHFNSKA**M**VIVVVNKG | KA**M**VIVVVNKGTGNLELVAV | KEGD**V**FIMPAAHPVAINASS |  |
| DRB1_0426 | FDQRSRQ**F**QNLQNHRIVQIE | NLQNHRIVQIEAKPNTLVLP | DLSNNFGKLFEVKPDKKNPQ | FEVKPDKKNPQLQDLDMMLT | LTCVEIKEGAL**M**LPHFNSKA | AL**M**LPHFNSKA**M**VIVVVNKG | KA**M**VIVVVNKGTGNLELVAV | KEGDVFIMPAAHPVAINASS |  |
| DRB1_0701 | FDQRSRQ**F**QN**L**QNHRIVQIE | N**L**QNHRIVQIEAKPNTLVLP | DLSNNFGKLFEVKPDKKNPQ | FEVKPDKKNPQLQDLDMMLT | LTCVEIKEGALMLPHFNSKA | ALMLPHFNSKA**M**VIVVVNKG | KA**M**VIVVVNKGTGNLELVAV | KEGDV**F**IMPAAHPVAINASS |  |
| DRB1_0703 | FDQRSRQ**F**QN**L**QNHRIVQIE | N**L**QNHRIVQIEAKPNTLVLP | DLSNNFGKLFEVKPDKKNPQ | FEVKPDKKNPQLQDLDMMLT | LTCVEIKEGALMLPHFNSKA | ALMLPHFNSKA**M**VIVVVNKG | KA**M**VIVVVNKGTGNLELVAV | KEGDV**F**IMPAAHPVAINASS |  |
| DRB1_0801 | **F**DQRSRQFQNLQNHRIVQIE | NLQNHRIVQIEAKPNTLVLP | DLSNNFGKLFEVKPDKKNPQ | FEVKPDKKNPQLQDLDMMLT | LTC**V**EIKEGALMLPHFNSKA | ALMLPHFNSKA**M**VIVVVNKG | KA**M**V**I**V**V**VNKGTGNLELVAV | KEGD**V**FIMPAAHPVAINASS |  |
| DRB1_0802 | **F**DQRSRQFQNLQNHRIVQIE | NLQNHRIVQIEAKPNTLVLP | DLSNNFGKLFEVKPDKKNPQ | FEVKPDKKNPQLQDLDMMLT | LTC**V**EIKEGALMLPHFNSKA | ALMLPHFNSKA**M**VIVVVNKG | KA**M**V**I**V**V**VNKGTGNLELVAV | KEGD**V**FIMPAAHPVAINASS |  |
| DRB1_0804 | FDQRSRQFQNLQNHRIVQIE | NLQNHRIVQIEAKPNTLVLP | DLSNNFGKLFEVKPDKKNPQ | FEVKPDKKNPQLQDLDMMLT | LTC**V**EIKEGALMLPHFNSKA | ALMLPHFNSKA**M**VIVVVNKG | KA**M**V**I**V**V**VNKGTGNLELVAV | KEGD**V**FIMPAAHPVAINASS |  |
| DRB1_0806 | FDQRSRQFQNLQNHRIVQIE | NLQNHRIVQIEAKPNTLVLP | DLSNNFGKLFEVKPDKKNPQ | FEVKPDKKNPQLQDLDMMLT | LTC**V**EIKEGALMLPHFNSKA | ALMLPHFNSKA**M**VIVVVNKG | KA**M**V**I**V**V**VNKGTGNLELVAV | KEGD**V**FIMPAAHPVAINASS |  |
| DRB1_0813 | **F**DQRSRQFQNLQNHRIVQIE | NLQNHRIVQIEAKPNTLVLP | DLSNNFGKLFE**V**KPDKKNPQ | FE**V**KPDKKNPQLQDLDMMLT | LTCVEIKEGALMLPHFNSKA | ALMLPHFNSKA**M**VIVVVNKG | KA**M**VIV**V**VNKGTGNLELVAV | KEGDVFIMPAAHPVAINASS |  |
| DRB1_0817 | FDQRSRQFQNLQNHRIVQIE | NLQNHRIVQIEAKPNTLVLP | DLSNNFGKLFEVKPDKKNPQ | FEVKPDKKNPQLQDLDMMLT | LTC**V**EIKEGALMLPHFNSKA | ALMLPHFNSKA**M**VIVVVNKG | KA**M**V**I**V**V**VNKGTGNLELVAV | KEGDVFIMPAAHPVAINASS |  |
| DRB1_1101 | FDQRSRQ**F**QNLQNHRIVQIE | NLQNHRIVQIEAKPNTLVLP | DLSNNFGKLFEVKPDKKNPQ | FEVKPDKKNPQLQDLDMMLT | LTCVEIKEGALMLPHFNSKA | ALMLPHFNSKA**M**VIVVVNKG | KA**M**V**I**VVVNKGTGNLELVAV | KEGDVFIMPAAHPVAINASS |  |
| DRB1_1102 | FDQRSRQFQNLQNHRIVQIE | N**L**QNHRIVQIEAKPNTLVLP | DLSNNFGKLFE**V**KPDKKNPQ | FE**V**KPDKKNPQLQDLDMMLT | LTCVEIKEGALMLPHFNSKA | ALMLPHFNSKA**M**VIVVVNKG | KA**M**VIVVVNKGTGNLELVAV | KEGD**V**FIMPAAHPVAINASS |  |
| DRB1_1104 | FDQRSRQFQNLQNHRIVQIE | NLQNHRIVQIEAKPNTLVLP | DLSNNFGKLFEVKPDKKNPQ | FEVKPDKKNPQLQDLDMMLT | LTCVEIKEGALMLPHFNSKA | ALMLPHFNSKA**M**VIVVVNKG | KA**M**V**I**VVVNKGTGNLELVAV | KEGD**V**FIMPAAHPVAINASS |  |
| DRB1_1106 | FDQRSRQFQNLQNHRIVQIE | NLQNHRIVQIEAKPNTLVLP | DLSNNFGKLFEVKPDKKNPQ | FEVKPDKKNPQLQDLDMMLT | LTCVEIKEGALMLPHFNSKA | ALMLPHFNSKA**M**VIVVVNKG | KA**M**V**I**VVVNKGTGNLELVAV | KEGD**V**FIMPAAHPVAINASS |  |
| DRB1_1107 | FDQRSRQFQN**L**QNHRIVQIE | N**L**QNHRI**V**QIEAKPNTLVLP | DLSNNFGKLFE**V**KPDKKNPQ | FE**V**KPDKKNPQLQDLDMMLT | LTCVEIKEGALMLPHFNSKA | ALMLPHFNSKA**M**VIVVVNKG | KA**M**V**I**VVVNKGTGNLELVAV | KEGDVFIMPAAHPVAINASS |  |
| DRB1_1114 | FDQRSRQ**F**QN**L**QNHRIVQIE | N**L**QNHRIVQIEAKPNTLVLP | DLSNNFGKLFEVKPDKKNPQ | FEVKPDKKNPQLQDLDMMLT | LTCVEIKEGALMLPHFNSKA | ALMLPHFNSKA**M**VIVVVNKG | KA**M**VIVVVNKGTGNLELVAV | KEGDVFIMPAAHPVAINASS |  |
| DRB1_1120 | FDQRSRQ**F**QN**L**QNHRIVQIE | N**L**QNHRIVQIEAKPNTLVLP | DLSNNFGKLFEVKPDKKNPQ | FEVKPDKKNPQLQDLDMMLT | LTCVEIKEGALMLPHFNSKA | ALMLPHFNSKA**M**VIVVVNKG | KA**M**VIVVVNKGTGNLELVAV | KEGDVFIMPAAHPVAINASS |  |
| DRB1_1121 | FDQRSRQFQN**L**QNHRIVQIE | N**L**QNHRIVQIEAKPNTLVLP | DLSNNFGKLFE**V**KPDKKNPQ | FE**V**KPDKKNPQLQDLDMMLT | LTCVEIKEGALMLPHFNSKA | ALMLPHFNSKA**M**VIVVVNKG | KA**M**VIVVVNKGTGNLELVAV | KEGD**V**FIMPAAHPVAINASS |  |
| DRB1_1128 | FDQRSRQ**F**QNLQNHRIVQIE | NLQNHRIVQIEAKPNTLVLP | DLSNNFGKLFEVKPDKKNPQ | FEVKPDKKNPQLQDLDMMLT | LTCVEIKEGALMLPHFNSKA | ALMLPHFNSKA**M**VIVVVNKG | KA**M**V**I**VVVNKGTGNLELVAV | KEGDVFIMPAAHPVAINASS |  |
| DRB1_1301 | FDQRSRQFQN**L**QNHRIVQIE | N**L**QNHRIVQIEAKPNTLVLP | DLSNNFGKLFEVKPDKKNPQ | FEVKPDKKNPQLQDLDMMLT | LTC**V**EIKEGALMLPHFNSKA | ALMLPHFNSKA**M**VIVVVNKG | KA**M**VIV**V**VNKGTGNLELVAV | KEGD**V**FIMPAAHPVAINASS |  |
| DRB1_1302 | FDQRSRQ**F**QN**L**QNHRIVQIE | N**L**QNHRIVQIEAKPNTLVLP | DLSNNFGKLFEVKPDKKNPQ | FEVKPDKKNPQLQDLDMMLT | LTCVEIKEGALMLPHFNSKA | ALMLPHFNSKA**M**VIVVVNKG | KA**M**VIVVVNKGTGNLELVAV | KEGDVFIMPAAHPVAINASS |  |
| DRB1_1304 | FDQRSRQFQN**L**QNHRIVQIE | N**L**QNHRIVQIEAKPNTLVLP | DLSNNFGKLFE**V**KPDKKNPQ | FE**V**KPDKKNPQLQDLDMMLT | LTCVEIKEGALMLPHFNSKA | ALMLPHFNSKA**M**VIVVVNKG | KA**M**VIV**V**VNKGTGNLELVAV | KEGDVFIMPAAHPVAINASS |  |
| DRB1_1305 | FDQRSRQ**F**QNLQNHRIVQIE | NLQNHRIVQIEAKPNTLVLP | DLSNNFGKLFEVKPDKKNPQ | FEVKPDKKNPQLQDLDMMLT | LTCVEIKEGALMLPHFNSKA | ALMLPHFNSKA**M**VIVVVNKG | KA**M**V**I**VVVNKGTGNLELVAV | KEGDVFIMPAAHPVAINASS |  |
| DRB1_1307 | FDQRSRQ**F**QNLQNHRIVQIE | NLQNHRIVQIEAKPNTLVLP | DLSNNFGKLFEVKPDKKNPQ | FEVKPDKKNPQLQDLDMMLT | LTCVEIKEGALMLPHFNSKA | ALMLPHFNSKA**M**VIVVVNKG | KA**M**V**I**VVVNKGTGNLELVAV | KEGD**V**FIMPAAHPVAINASS |  |
| DRB1_1311 | FDQRSRQFQNLQNHRIVQIE | NLQNHRIVQIEAKPNTLVLP | DLSNNFGKLFEVKPDKKNPQ | FEVKPDKKNPQLQDLDMMLT | LTCVEIKEGALMLPHFNSKA | ALMLPHFNSKA**M**VIVVVNKG | KA**M**V**I**VVVNKGTGNLELVAV | KEGD**V**FIMPAAHPVAINASS |  |
| DRB1_1321 | FDQRSRQFQNLQNHRIVQIE | NLQNHR**I**VQIEAKPNTLVLP | DLSNNFGKLFEVKPDKKNPQ | FEVKPDKKNPQLQDLDMMLT | LTCVEIKEGALMLPHFNSKA | ALMLPHFNSKA**M**VIVVVNKG | KA**M**V**I**VVVNKGTGNLELVAV | KEGDVFIMPAAHPVAINASS |  |
| DRB1_1322 | FDQRSRQFQN**L**QNHRIVQIE | N**L**QNHRIVQIEAKPNTLVLP | DLSNNFGKLFE**V**KPDKKNPQ | FE**V**KPDKKNPQLQDLDMMLT | LTCVEIKEGALMLPHFNSKA | ALMLPHFNSKA**M**VIVVVNKG | KA**M**VIVVVNKGTGNLELVAV | KEGD**V**FIMPAAHPVAINASS |  |
| DRB1_1323 | FDQRSRQ**F**QN**L**QNHRIVQIE | N**L**QNHRIVQIEAKPNTLVLP | DLSNNFGKLFEVKPDKKNPQ | FEVKPDKKNPQLQDLDMMLT | LTCVEIKEGALMLPHFNSKA | ALMLPHFNSKA**M**VIVVVNKG | KA**M**VIVVVNKGTGNLELVAV | KEGDVFIMPAAHPVAINASS |  |
| DRB1_1327 | FDQRSRQFQN**L**QNHRIVQIE | N**L**QNHRIVQIEAKPNTLVLP | DLSNNFGKLFEVKPDKKNPQ | FEVKPDKKNPQLQDLDMMLT | LTC**V**EIKEGALMLPHFNSKA | ALMLPHFNSKA**M**VIVVVNKG | KA**M**VIV**V**VNKGTGNLELVAV | KEGD**V**FIMPAAHPVAINASS |  |
| DRB1_1328 | FDQRSRQFQN**L**QNHRIVQIE | N**L**QNHRIVQIEAKPNTLVLP | DLSNNFGKLFEVKPDKKNPQ | FEVKPDKKNPQLQDLDMMLT | LTC**V**EIKEGALMLPHFNSKA | ALMLPHFNSKA**M**VIVVVNKG | KA**M**VIV**V**VNKGTGNLELVAV | KEGD**V**FIMPAAHPVAINASS |  |
| DRB1_1501 | FDQRSRQFQN**L**QNHRIVQIE | N**L**QNHRIVQIEAKPNTLVLP | DLSNNFGKLFEVKPDKKNPQ | FEVKPDKKNPQLQDLDMMLT | LTCVEIKEGAL**M**LPHFNSKA | AL**M**LPHFNSKA**M**VIVVVNKG | KA**M**VIVVVNKGTGNLELVAV | KEGDVFIMPAAHPVAINASS |  |
| DRB1_1502 | FDQRSRQ**F**QNLQNHRIVQIE | NLQNHRIVQIEAKPNTLVLP | DLSNNFGKLFEVKPDKKNPQ | FEVKPDKKNPQLQDLDMMLT | LTCVEIKEGALMLPHFNSKA | ALMLPHFNSKAMVIVVVNKG | KAMVIVVVNKGTGNLELVAV | KEGDVFIMPAAHPVAINASS |  |
| DRB1_1506 | FDQRSRQ**F**QN**L**QNHRIVQIE | N**L**QNHRIVQIEAKPNTLVLP | DLSNNFGKLFEVKPDKKNPQ | FEVKPDKKNPQLQDLDMMLT | LTCVEIKEGAL**M**LPHFNSKA | AL**M**LPHFNSKA**M**VIVVVNKG | KA**M**VIVVVNKGTGNLELVAV | KEGDVFIMPAAHPVAINASS |  |
| DRB5_0101 | FDQRSRQ**F**QNLQNHRIVQIE | NLQNHR**I**VQIEAKPNTLVLP | DLSNNFGKLFEVKPDKKNPQ | FEVKPDKKNPQLQDLDMMLT | LTCVEIKEGALMLPHFNSKA | ALMLPHFNSKA**M**VIVVVNKG | KA**M**VIVVVNKGTGNLELVAV | KEGDVFIMPAAHPVAINASS |  |
| DRB5_0105 | FDQRSRQ**F**QNLQNHRIVQIE | NLQNHR**I**VQIEAKPNTLVLP | DLSNNFGKLFEVKPDKKNPQ | FEVKPDKKNPQLQDLDMMLT | LTCVEIKEGALMLPHFNSKA | ALMLPHFNSKA**M**VIVVVNKG | KA**M**VIVVVNKGTGNLELVAV | KEGDVFIMPAAHPVAINASS |  |

HLA-DR binding motifs (grey shading) were predicted using the ProPred algorithm [1], (http://www.immuneepitope.org; accessed 30^th^ January 2012). Predicted primary anchor residues are bolded and underlined. Peptide 40 (352-371) is not shown as no HLA-DR binding motifs were predicted for this peptide by this algorithm

**Figure Legends:**

**Figure S1: Representative CFSE-based assay for detecting CD4+ T-cell proliferation in PBMC**

Proliferation of CFSE-labelled PBMC from peanut-allergic subject 26 following 7 days stimulation with selected Ara h 1 20-mer peptides. Medium alone (No Antigen) or crude peanut extract (CPE) provided negative and positive controls respectively. At least 10,000 live CD4^+^ T cells were analyzed per sample. Gates indicate percentage CD4^+^CFSE^lo^ (proliferating) T cells of total CD4^+^ T cells with stimulation indices (SI) in parentheses.

**Figure S2: Representative HLA class II restriction specificity of T-cell epitope recognition**

Proliferation of specific TCL to selected epitopes in the presence of HLA-DR (circles), -DQ (squares) or -DP (triangles) mAbs (Ai and Bi) or isotype control antibodies (10 μg/ml) (Aii and Bii), (mean cpm replicate wells +SD). Graphs show sample data for an HLA-DR-restricted epitope (442-458) (A) and an HLA-DQ restricted epitope (507-524) (B). **References:**

1. Singh H, Raghava GP. ProPred: prediction of HLA-DR binding sites. Bioinformatics. 2001;17(12):1236-7.
